# The Synthesis and Photophysical Properties of Weakly Coupled Diketopyrrolopyrroles

**DOI:** 10.3390/molecules26164744

**Published:** 2021-08-05

**Authors:** Michał Pieczykolan, James B. Derr, Amara Chrayteh, Beata Koszarna, John A. Clark, Olena Vakuliuk, Denis Jacquemin, Valentine I. Vullev, Daniel T. Gryko

**Affiliations:** 1 Institute of Organic Chemistry, Polish Academy of Sciences, Kasprzaka 44-52, 01-224 Warsaw, Poland; Michal.Pieczykolan@rwth-aachen.de (M.P.); beata.koszarna@icho.edu.pl (B.K.); olena.vakuliuk@icho.edu.pl (O.V.); 2 Department of Biochemistry, University of California, Riverside, CA 92521, USA; jderr002@ucr.edu; 3 CEISAM Laboratory—UMR 6230, University of Nantes, CNTS, 44035 Nantes, France; amara.chrayteh@univ-nantes.fr; 4 Department of Bioengineering, University of California, Riverside, CA 92521, USA; jclar019@ucr.edu

**Keywords:** dyes, fluorescence, direct arylation, diketopyrrolopyrroles

## Abstract

Three centrosymmetric diketopyrrolopyrroles possessing either two 2-(2′-methoxyphenyl)benzothiazole or two 2-(2′-methoxyphenyl)benzoxazolo-thiophene scaffolds were synthesized in a straightforward manner, and their photophysical properties were investigated. Their emission was significantly bathochromically shifted as compared with that of simple DPPs reaching 650 nm. Judging from theoretical calculations performed with time-dependent density functional theory, in all three cases the excited state was localized on the DPP core and there was no significant CT character. Consequently, emission was almost independent of solvents’ polarity. DPPs possessing 2,5-thiophene units vicinal to DPP core play a role in electronic transitions, resulting in bathochromically shifted absorption and emission. Interestingly, as judged from transient absorption dynamics, intersystem crossing was responsible for the deactivation of the excited states of DPPs possessing *para* linkers but not in the case of dye bearing *meta* linker.

## 1. Introduction

In the chemistry of dyes and pigments, systems with extended π-conjugation have prevailed in the literature for years [1,2,3,4,5,6,7,8]. There is, however, an increased interest in weakly coupled systems containing multiple biaryl linkages [9,10,11,12,13,14]. Depending on the dihedral angle both in the ground and in the excited states, the magnitude of electronic communication varies, leading to large Stokes’ shifts and strong solvatochromism of fluorescence. In particular, modulations of the planarization and polarization of such chromophores with push–pull configurations has become a conceptually new approach towards modern fluorescent probes. They exhibit sensitivity not only to solvent polarity but also to medium viscosity. Matile and co-workers have shown that such probes respond to changes in the fluidity of lipid bilayer membranes [15,16,17,18,19,20].

**Figure 1:**
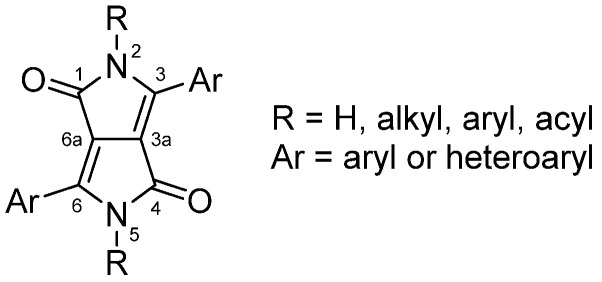
General structure of diketopyrrolopyrroles with atom numeration.

Looking for chromophores, which possess the suitable architecture for probing via weakening of their intramolecular electronic coupling, we focused on diketopyrrolopyrrole (DPP) derivatives (Figure 1) [21,22,23,24,25,26,27,28,29]. These donor–acceptor cross-conjugated dyes with DPP cores are characterized by straightforward synthesis [30,31,32,33,34,35,36,37], almost a unity emission quantum yield [21], large two-photon absorption cross-section [38,39,40,41,42,43,44,45], and broad utility in organic optoelectronics [46,47,48,49,50,51,52,53,54,55,56,57,58,59,60,61,62,63,64,65,66,67,68,69,70,71]. Depending on the dihedral angle between *C*-aryl substituents and the core, the absorption of DPPs ranges from 500 to 600 nm [21]. The relationship between the structure of the DPPs and their linear and non-linear optical properties has been probed in numerous studies [72,73,74,75,76,77,78]. Replacing the phenyls with aromatic five-membered rings at positions 3 and 6 in *N*,*N′*-dialkylated DPPs decreased the dihedral angle from ca. 30 to ~7 degrees, thus altering the photophysical properties [21]. In particular, a bathochromic shift of absorption is often observed [79,80,81,82], accompanied by better packing in the crystalline state, stronger charge transfer, and larger two-photon absorption cross-sections [38,39,40,41,42,43,44,45]. It is, therefore, interesting to study multiple architecture based on the DPP scaffold-possessing groups such as benzoxazole and benzothiazole either directly linked via benzene ring or elongated via thiophene rings.

## 2. Results

### 2.1. Design and Synthesis

**Scheme 1:**
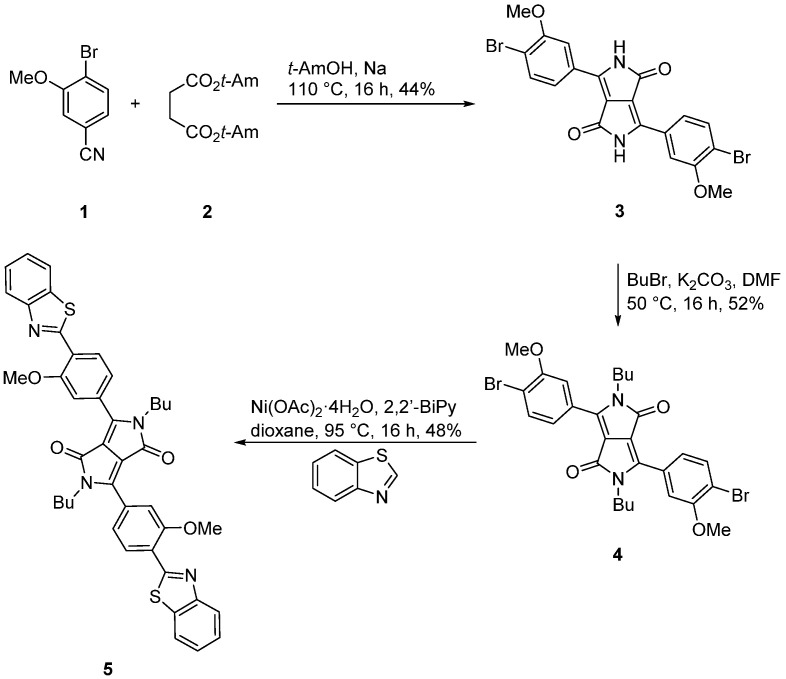
The synthesis of diketopyrrolopyrrole **5**.

**Scheme 2:**
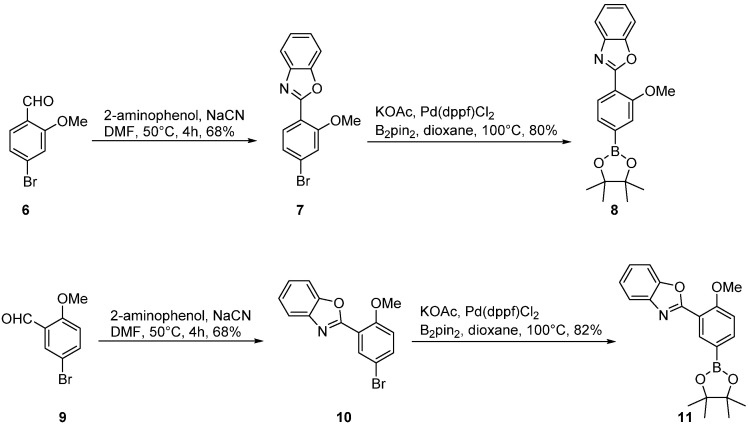
The synthesis of benzoxazoles **8** and **11**.

**Scheme 3:**
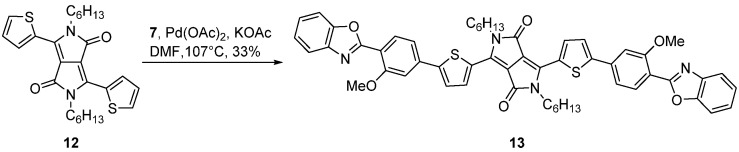
The synthesis of DPP **13**.

**Scheme 4:**
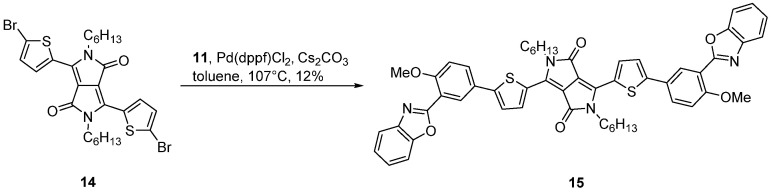
The synthesis of DPP **15**.

Our design of *N*,*N*′-dialkyldiketopyrrolopyrroles was based on placing two different rings between benzoxazole/benzothiazole units and DPP core. In the first case, we bridged them with 1,4-phenylene linkers (**5**, Scheme 1). An addition of 2,5-thiophen units between the DPP core and the benzene rings (**13** and **15**, Scheme 2, Scheme 3 and Scheme 4), while extending the linkers, enhanced the electronic communication by decreasing the dihedral angles. The benzoxazole unit and methoxy unit were alternatingly placed at positions 1,4 and 1,3.

Therefore, we initiated our investigation with the preparation of DPP **3**, which contains two 3-methoxyphenyl substituents. We recently learned that if classical diisopropyl succinate is used in reactions with 3,4-dimethoxybenzonitryle, the replacement of one or more methoxy groups with isopropyl groups is a side reaction. Therefore, we resolved this by using more sterically hindered bis(*tert*-amyl)succinate (**2**) in reaction with nitrile **1** (Scheme 1). The pigment **3** was subsequently *N*-alkylated under classical conditions to give DPP **4** in 23% overall yield (Figure S1). DPP **4** was subsequently used as arylating agent in direct arylation of benzothiazole under recently described conditions [83], leading to the formation of DPP **5** (Scheme 1, Figure S2).

Independently, two regioisomeric methoxybenzaldehydes **6** and **9** possessing bromine atom were condensed with 2-aminophenol to give corresponding benzoxazoles **7** and **10** (Scheme 2, Figures S3 and S5). Subsequently bromine atoms were replaced with pinacolo-borane to give highly functionalized heterocyclic building blocks **8** and **11** (Figures S4 and S6).

Interestingly, the synthesis of DPP **13** from DPP **14** and **8** via Suzuki coupling led to the formation of dye **13** in 1% yield only. Therefore, we resorted to direct arylation, which had recently become a major tool in the synthesis of dyes [84,85] (including DPPs) [86,87,88], and heterocyclic PAHs [89]. Gratifyingly, DPP **12** underwent smooth reaction with compound **7** under the reaction conditions developed by Doucet and co-workers [90], to afford the desired DPP **13** in 33% yield (Scheme 3, Figure S7). In contrast to that result, the Suzuki coupling of DPP **14** with benzoxazole **11** gave DPP **15** in reasonable yield (Scheme 4, Figure S8).

### 2.2. Photophysical Properties

**Figure 2:**
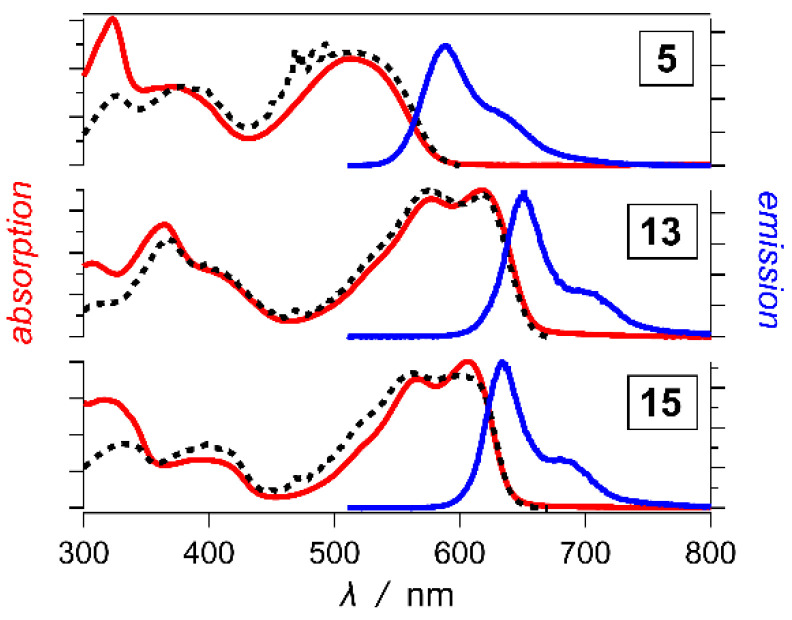
Absorption (red), excitation (dotted black), and emission (blue) spectra of compounds **5, 13**, and **15** for DCM.

For the photophysical studies of diketopyrrolopyrroles **5**, **13**, and **15** using steady-state and time-resolved optical spectroscopy, we employed toluene (TOL) (Figure S9), dichloromethane (DCM), and *N*,*N*-dimethylformamide (DMF) (Figure S10) as solvents (Table 1, Figure 2). The red solutions of **5** showed fluorescence quantum yields (Φ_fl_) ranging between 0.7 and 0.9 and excited-state lifetimes (*τ*) around 4 ns, which is on par with what is expected for DPP chromophores (Table 1). The Stokes’ shifts of about 2000 cm^−1^ suggest that the chromophore was exhibiting some changes in the geometry between the Franck–Condon and the fluorescent excited state. The lack of polarity dependence of *τ* and of the spectral maxima, *λ*_abs_ and *λ*_fl_, precluded transitions involving excited states with a pronounced charge-transfer (CT) character. The 20% decrease in Φ_fl_ of **5** upon transitioning to the polar DMF medium, while noticeable, cannot be ascribed to intramolecular excited-state CT. Dyes **5**, **13**, and **15** showed propensity for aggregation in solvents different from DCM. They all formed clear solution, but broad weak absorption bands in the near-infrared (NIR) region indicate ground-state aggregation. For **13** and **15**, the ground-state aggregation was noticeable for toluene and especially apparent for DMF. While the lifetimes extracted from the emission decays accounted only for the state responsible for the fluorescence around 600–700 nm, the absorption was cumulative for all species, resulting in the slightly decreased values of Φ_fl_ for DMF.

Introducing thiophene linker between the DPP core and 2-(2′-methoxyphenyl) moieties resulted in bathochromic shifts of the first absorption maximum from about 510–530 nm for **5** to 610–620 nm for **13** and **15** (Table 1, Figure 2). These shifts were more pronounced for the *para*-benzoxazole derivative, **13**, than for the *meta*-one, **15**. This feature is consistent with improving the electronic coupling across a phenylene linker upon shifting the substituents from *meta* to *para* position. The fluorescence maxima showed the same trends of bathochromic shifts upon extending the linker and moving the benzoxazole units from the *meta* to the *para* sites (Table 1, Figure 2).

In the case of DPPs **13** and **15,** a vibronic structure of the absorption band emerged, and their Stokes’ shifts decreased to 800 cm^−1^. The fluorescence quantum yields of **13** and **15** decreased by about a factor of three in comparison with Φ_fl_ of **5**; and the excited-state lifetimes of **13** and **15** decreased by about 25% (Table 1). They indicate a 2-fold decrease in the radiative decay rate constants, *k_f_*, of **13** and **15** that accompanied a 7-fold increase in the non-radiative decay rate constants, *k_nr_* (Table 1).

Extending the π-conjugation and switching from benzothiazole of **5** to the more electron-withdrawing benzoxazole of **13** and **15** can decrease the overlaps between the natural transition orbitals (NTOs) for the S_1_→S_0_ radiate transitions responsible for the decrease in *k_f_*. At the same time, the extra dihedral degrees of freedom in the aromatic linkers of **13** and **15** offer additional configurations that can bring the potential-energy surfaces (PESs) of the S_1_ and S_0_ states close to each other, providing internal conversion (IC) pathways for efficient non-radiative deactivation, as the increase in *k_nr_* reflects (Table 1).

While time-correlated single-photon counting (TCSPC) allows probing the dynamics of fluorescent photoexcited species, it is too slow for fast picosecond transitions along the S_1_ PES and cannot monitor any dark states that may form. Therefore, we resorted to pump-probe transient-absorption TA spectroscopy to elucidate further the excited-state dynamics of these DPPs.

**Figure 3:**
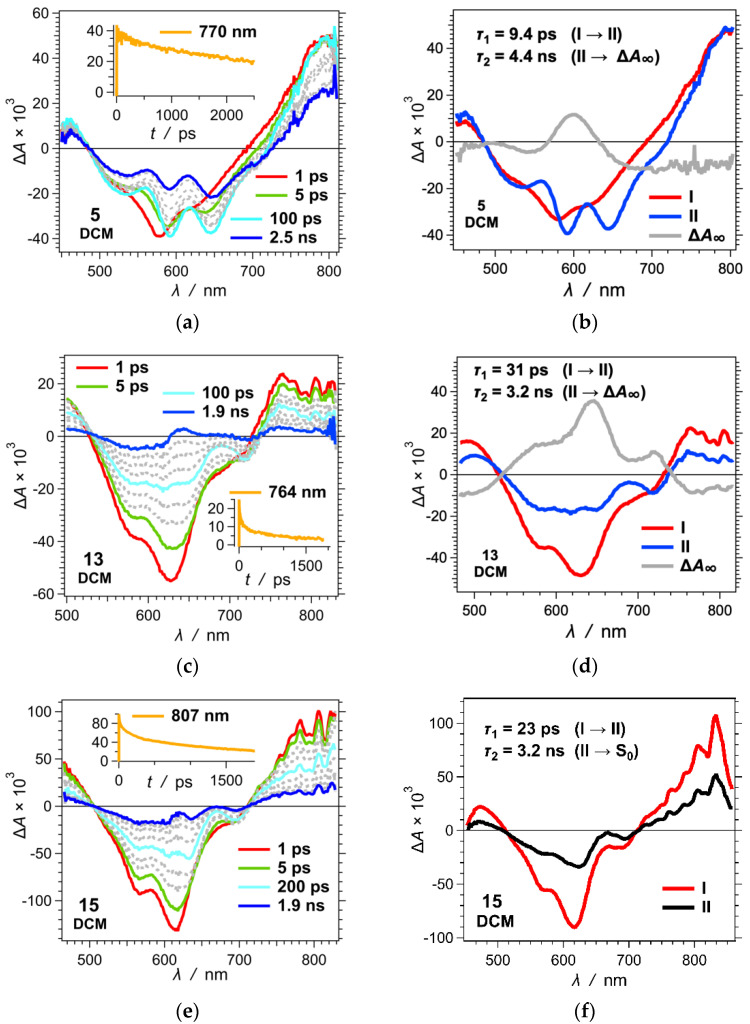
Transient-absorption dynamics of compounds **5**, **13**, and **15** for DCM. (**a**,**c**,**e**) Representative TA spectra of **5**, **13**, and **15** for DCM. Insets: the decays of the singlet excited states, ^1^DPP*, of **5**, **13**, and **15** (*λ_ex_* = 400 nm, 5 μJ per pulse). (**b**,**d**,**f**) Spectra of **5, 13,** and **15** for DCM, representing the transitions associated with each of the time constants, *τ_i_*, obtained from the decay associated difference spectra, *α_i_* vs. *λ*, that biexponential global fits produce, i.e., Δ*A*(*λ*, *t*) = Δ*A*_∞_(*λ*) + *α*_1_(*λ*) exp(−*t/τ*_1_) + *α*_2_(*λ*) exp(−*t/τ*_2_). (I and II—transition spectra; Δ*A*_∞_—spectra of transients with lifetimes that considerably exceeds the dynamic range of the pump-probe technique; S_0_—ground state). The nanosecond time constants, *τ*_2_, are obtained from TCSPC and introduced as set parameters to the global fit analysis.

The TA spectra show the ground state bleach at 550–650 nm, modest stimulated emission at 695 nm, and the absorption of the singlet excited state of DPP at 750–800 nm (Figure 3a,c,e) [91,92,93]. In addition to these principal features that were to be expected for DPP conjugates, the evolution of the TA spectra revealed a subtle rise of absorption bands in the region around 550 to 650 nm (Figure 3a,c,e). This week TA band at 640 nm became especially apparent for **13** at nanosecond probe delays (Figure 3c).

Global fit (GF) analysis revealed that, prior to the nanosecond decays that TCSPC showed, the three DPPs underwent picosecond transitions (Figure 3b,d,f). For **5**, the 10 ps transition led to shifts of some of the band and reshaping the TA spectra without significant change in the amplitudes (Figure 3b). The nanosecond decay led to a small TA band centered around 600 nm, indicating the formation of a transient with a lifetime quite longer than the dynamic range of the pump-probe technique, as represented by Δ*A*_∞_ (Figure 3b).

In contrast, a decrease of the TA amplitudes by about a factor of two reflected the picosecond transitions of the conjugates with thiophene moieties **13** and **15** (Figure 3d,f). Allowing Δ*A*_∞_(*λ*) to fluctuate for **13**, and setting Δ*A*_∞_(*λ*) = 0 for **15**, aided the conversion of the GFs. That is, the nanosecond decay of **13** led to a long-lived transient, with TA peaking at about 645 nm (Figure 3d). While **15** also may form such a long-lived transient, its TA amplitudes were undetectable by the GF analysis.

The DPP radical anion had a pronounced TA band at around 640 nm [91,93]. Nevertheless, the triplets of different DPP conjugates also absorbed between about 600 and 650 nm [94,95]. Considering the picosecond intramolecular charge transfer (CT) of other DPP conjugates, followed by subnanosecond back CT [91,92,93], suggests that the transients revealed by Δ*A*_∞_(*λ*) formed too slowly and had too long lifetimes to have had originated from the radical anion of DPP. Therefore, the long-lived transients that **5** and **13** formed most plausibly indicates for triplet formation. The TA dynamics suggests that intersystem crossing (ISC) principally contributed to the deactivation of the excited states of **5** and **13**. In the excited-state dynamics of **15**, on the other hand, ISC did not play a key role.

### 2.3. Theoretical Modelling

Time-dependent density-functional theory (TD-DFT) calculations allowed us to explore the structural and optical properties of the investigated dyes. The ground-state geometry of **5** showed the expected dihedral angles between the DPP core and the benzene rings (31°) and between these benzene rings and the benzothiazole moieties (39°), the presence of the methoxy substituents preventing planarity for the latter. There was a partial planarization in the lowest excited state with respective dihedrals of 20° and 19°, respectively. In contrast, **13** and **15** with the additional thienyl spacer allowed for a stronger π-conjugation, with dihedrals between the thienyl groups and the DPP core of 2° (1°) in the ground-state and 0° (1°) in the lowest excited-state. Nevertheless, the planarization of the benzene and benzoxazole upon going from the ground to the excited state pertained to both **13** and **15**, with, e.g., a thienyl-phenyl twist going from 26° to 4° in **13**.

**Figure 4:**
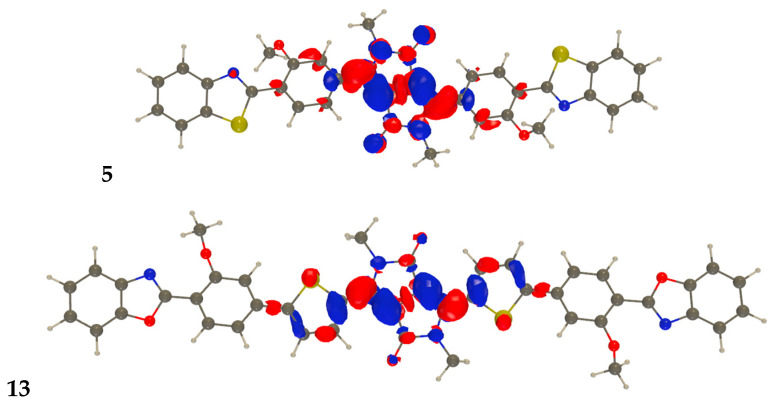

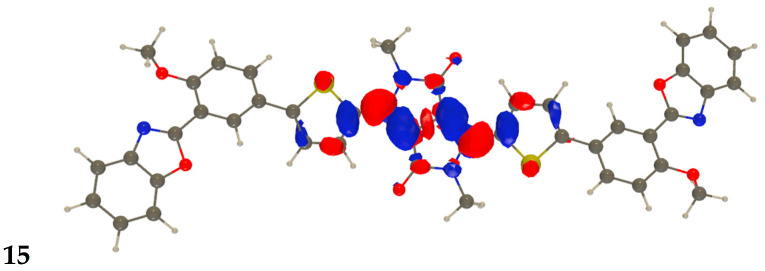
Density difference plots for the lowest absorption of (from top to bottom): **5**, **13**, and **15**. The crimson and blueberry regions indicate zones of increase and decrease of electronic density upon photoabsorption. A contour of 1 × 10^−3^ au is used.

Theory returned 0-0 wavelengths of 556, 646, and 629 nm for **5**, **13**, and **15** in DCM, respectively. These values compare favorably with the experimental position of the crossing point between the absorption/emission curves, i.e., 515, 631, and 620 nm (Figure 2). These results show that theory reproduced both quantitatively and qualitatively the experimental values. Electron density difference (EDD) plots provide representation of the transitions between the ground and excited states (Figure 4). The excited state was systematically localized on the DPP core with no significant CT character, which is consistent with the lack of solvent-polarity effects on the experimentally obtained absorption and emission maxima (Table 1). It further confirms that the long-lived transients of **5** and **13**, which their TA spectra revealed (Figure 3b,d), are their triplets, rather than long-lived CT states.

Indeed, the amount of transferred charged *q*^CT^ amounted to ca. 0.3–0.4 e in all three dyes, clearly confirming the localized nature of the transition. Nevertheless, EDD of **5** indicates that the side benzene rings played a trifling role in the optical transitions (Figure 4). The EDDs of **13** and **15** on the other side, on the other hand, reveal that the thienyl moieties vicinal to the DPP core contributed to the optical transitions (Figure 4), which is consistent with their bathochromically shifted absorption (Figure 2, Table 1). This extension of the EDD was slightly more pronounced for the *para*-benzoxazole derivative, **13**, than with the *meta*-one, **15** (Figure 4), which agrees with what the absorption spectra show (Figure 2).

## 3. Discussion

The decrease in dihedral angle upon moving from phenyl substituents (~30°) to 5-membered rings (~7°) leads to significant changes in the photophysical properties of the resultant *N*,*N*-dialkylDPPs, with an especially notable bathochromic shift in absorption [21,22,23,24,25,26,27,28,29]. The lower dihedral angle allows the substituent to have a greater contribution to the π-system of the DPP core in the ground state [21,22,23,24,25,26,27,28,29]. Our study corroborates these earlier observations, i.e., the replacement of 1,4-phenylene with a 2,5-thienylene as a linker directly attached to DPP core (**5** vs. **13**, **15**) leads to a 100 nm red-shift of both absorption and emission (Table 1). These properties resemble a DPP possessing two pyrene-2-yl-thienyl substituents [80]. This π-expansion leads to drastic decrease in the fluorescence quantum yields from what has been previously observed by Würthner and co-workers [79].

Moving the benzoxazole moiety from position 4 to position 3 of the benzene ring (**13**→**15**) slightly alters the photophysical properties of the resulting DPP. Both the absorption and emission of **15** are shifted hypsochromically (~10 nm), and increased radiative constants are observed in all solvents studied (Table 1).

## 4. Materials and Methods

All commercial materials (Sigma-Aldrich, Fluorochem, etc.) were used without further purification. All solvents were reagent or HPLC (Honeywell) grade. Unless otherwise noted, all reactions were run under argon atmosphere in flame-dried glassware. Reactions were stirred using Teflon-coated magnetic stir bars. TLC was performed on aluminium sheets, Merck 60F with fluorescent indicator F254 (Merck, Darmstadt, Germany). Plates were visualized by treatment with UV, acidic *p*-anisaldehyde stain, KMnO_4_ stain, or aqueous ceric ammonium molybdate (Hanessian’s stain; CAM) with gentle heating. Products were purified by flash column chromatography using the solvent systems indicated. Column chromatography was performed on Merck silica gel 60, 230–400 mesh.

All reported ^1^H-NMR spectra were recorded using Bruker 400 MHz (Bruker BioSpin GmbH, Rheinstetten, Germany) or Varian 500 or 600 MHz (Agilent, Santa Clara, CA 95051, United States) spectrometers. Chemical shifts are quoted on the (*δ* ppm) scale, multiplicity (s = singlet, bs = broad singlet, d = doublet, t = triplet, q = quartet, m = multiplet), coupling constants are given in Hz. Solvent signal was indicated as the internal standard (CDCl_3_, ^1^H-NMR 7.26 ppm)^1^ unless otherwise noted; *J* values are given in Hz. The mass spectra were obtained via electron ionization (EI-MS). Diketopyrrolopyrroles **12** and **14** were prepared following the literature procedure [96].

Steady-state absorption spectra were recorded in a transmission mode using a JASCO V-670 spectrophotometer (Jasco, Tokyo, Japan). The steady-state emission spectra and the time-correlated single-photon counting (TCSPC) fluorescence decays were measured using a FluoroLog-3 spectrofluorometer (Horiba-Jobin-Yvon, Edison, NJ, USA) equipped with a pulsed diode laser (λ = 406 nm, 196 ps pulse width) as previously reported. The wavelengths of the maxima of the absorption and emission spectra were obtained from fitting the spectra peaks with Gaussian function. For estimating zero-to-zero energy, E_00_, of a conjugate, we plotted its absorption and fluorescence spectra on the same graph where the fluorescence maximum was adjusted to be equal to the maximum of the band at the red edge of the absorption spectrum. E_00_ was estimated from the wavelength at which the thus normalized spectra crossed. The fluorescence quantum yields, Φ_fl_, were determined by comparing the integrated emission intensities of the samples with the integrated fluorescence of a reference sample with a known fluorescence quantum yield, where F(λ) is the fluorescence intensity at wavelength λ; A(λ_ex_) is the absorbance at the excitation wavelength; n is the refractive index of the media; and the suffix “0” indicates the quantities for the reference sample used. For a reference sample we used an aqueous solution of fluorescein buffered at pH 10 (Φ_fl_ = 0.93).

The transient-absorption (TA) data, Δ*A*(λ, t), were recorded in transmission mode with 2 mm quartz cuvettes using a Helios pump-probe spectrometer (Ultrafast Systems, LLC, Sarasota, FL, USA) equipped with a delay stage allowing maximum probe delays of 3.2 ns at 7 fs temporal step resolution [16]. Following chirp correction, we extracted the TA spectra and decays from Δ*A*(λ, t). The absorbance of the samples at the excitation wavelength, *A*(λ_ex_), was adjusted to about 0.4 to 0.6 in the 2 mm cuvettes. Immediately prior to the measurements, all samples were purged with argon for 5 to 10 min per 1 mL of sample. The photostability of the samples during the exposure to the pump laser was confirmed by comparing the absorption spectra recorded before and after each set of TA measurements. The laser source for the Helios was a SpitFire Pro 35F regenerative amplifier (Spectra Physics, Newport, CA, USA) generating 800 nm pulses (>35 fs, 4.0 mJ, at 1 kHz). The amplifier was pumped with of an Empower 30 Q-switched laser that ran at 20 W at the 2nd harmonic. A MaiTai SP oscillator provided the seed beam (55 nm bandwidth). The wavelength of the pump was tuned using an optical parametric amplifier, OPA-800CU (Newport Corporation, Newport, CA, USA), equipped with reflectors for removing the 800 nm fundamental and the signal, and the idler was subjected to second and fourth harmonic generators. For optimal OPA performance, the pulse duration from the amplifier was tuned to 50 fs. The idler was tuned in the range between 1840 and 1940 nm for selective excitation of DPP chromophore after upconversion to the fourth harmonic. The power of the signal and the idler removing the removal of the fundamental was stabilized at about 170 mW.

The fluorescence quantum yields, Φ*_fl_*, were estimated using fluorescein in aqueous alkaline solution as a standard:

Φ*_fl_*/Φ*_fl_*^(0)^ = (*F/F*^(0)^) ((1 − 10^−*A*(0)^)/(1 − 10^−*A*^)) (*n/n*^(0)^)^2^

where *A* is the absorbance (in the range of 0.01–0.15), *F* is the area under the curves of the emission spectra, *n* is the refractive index of the solvents (at 25 °C) used in measurements, and the superscripts (0) designates the standard. The following refractive index values were used: 1.424 for DCM, 1.344 for MeCN, 1.477 for DMSO, 1.362 for EtOH, 1.33 for aqueous 0.1 N NaOH.

*3,6-Bis(4-bromo-3-methoxyphenyl)-2,5-dibutyl-2,5-dihydropyrrolo [3,4-c]pyrrole-1,4-dione* (**4**). Sodium (0.92 g, 40.0 mmol), catalytic amount of FeCl_3_, and *tert-*amylalcohol (20.0 mL) were added to a 100 mL dried flask at room temperature under argon gas. Then the reaction mixture was stirred for 2 h at 95 °C until sodium dissolved. After cooling the mixture to 60 °C, nitrile **1** (2.12 g, 10.0 mmol) was added in one portion, temperature was again raised up to 110 °C, and diisopropyl succinate (1.01 g, 5.0 mmol) dissolved in *tert-*amylalcohol (2 mL) was added dropwise during next hour. The mixture was heated to 110 °C and stirred at the temperature for 20 h. After cooling the mixture to 60 °C, acetic acid (2 mL) and methanol (40 mL) were added, and the mixture was stirred at 60 °C for 30 min, cooled to room temperature, and then poured into methanol (100 mL). Red insoluble precipitate was formed, filtered, and washed with fresh portion (50 mL) of cold MeOH. Crude material (1.66 g) was suspended in DMF 30 mL, K_2_CO_3_ (2.76 g, 20.0 mmol) was added in one portion, and reaction mixture was stirred in 50 °C for 2 h; next, 1-bromobutane (4.11 g, 30 mmol) was added. Temperature was increased to 70 °C, and the mixture was stirred overnight. Solvent was evaporated, and crude material was purified by flash column chromatography (silica gel, EtOAc/hexanes, 3:7) to give **4** as red shiny crystals 710 mg (23%). ^1^H-NMR (400 MHz, CDCl_3_) δ 7.67 (d, *J* = 8.2 Hz, 2H), 7.62 (d, *J* = 2.0 Hz, 2H), 7.17 (dd, *J* = 8.2, 2.0 Hz, 2H), 4.03 (s, 6H), 3.82–3.77 (m, 4H), 1.59 (quin, *J* = 7.6 Hz, 4H), 1.28–1.31 (m, 4H), 0.87 (t, *J* = 7.6 Hz, 6H). ^13^C-NMR (151 MHz, CDCl_3_) δ 162.5, 156.3, 147.5, 133.6, 128.4, 120.9, 115.3, 112.8, 110.0, 56.6, 41.9, 31.6, 20.0, 13.6. HRMS (EI *m*/*z*): [M^+•^] Calcd. for C_28_H_30_N_2_O_4_Br_2_: 616.0572; found, 616.0564.

*3,6-Bis(4-(benzo[d]thiazol-2-yl)-3-methoxyphenyl)-2,5-dibutyl-2,5-dihydropyrrolo [3,4-c]pyrrole-1,4-dione* (**5**). A 50 mL Ace pressure vessel containing Ni(OAc)_2_·4H_2_O (249 mg, 1.0 mmol) was dried with a heat gun under vacuum and filled with argon after cooling to room temperature. To this vessel were added bipy (156 mg, 1.0 mmol), LiO*t*-Bu (24.0 mg, 0.30 mmol), compound **4** (61.8 mg, 0.10 mmol), and benzothiazole (40.6 mg, 0.30 mmol), followed by dry 1,4-dioxane (25 mL). The resulting mixture was heated at 95 °C for 16 h with stirring. After cooling to room temperature, solvent was evaporated and crude material was purified by flash column chromatography (silica gel, EtOAc/hexanes, 3:7) to give 35 mg (48%) of **5** as red shiny crystals. ^1^H-NMR (500 MHz, CDCl_3_) δ 8.47 (d, *J* = 8.3 Hz, 2H), 8.20 (d, *J* = 8.3 Hz, 2H), 8.08 (d, *J* = 8.3 Hz, 2H), 7.92 (s, 2H), 7.85 (t, *J* = 7.8 Hz, 2H), 7.76 (t, *J* = 7.8 Hz, 2H), 7.56 (d, *J* = 7.9 Hz, 2H), 4.32 (br s, 6H), 3.88–3.85 (m, 4H), 1.62–1.58 (m, 4H), 1.28–1.31 (m, 4H), 0.87 (t, *J* = 7.6 Hz, 6H). ^13^C-NMR (126 MHz, CDCl_3_) δ 166.3, 162.9, 158.6, 148.7, 139.6, 135.0, 130.7, 130.4, 129.9, 129.0, 122.5, 121.0, 118.2, 116.6, 113.8, 111.8, 57.2, 42.7, 31.4, 19.7, 13.2. HRMS (EI *m*/*z*): [M^+•^] Calcd. for C_42_H_38_N_4_O_4_S_2_: 726.2334; found, 726.2395.

*2-(4-Bromo-2-methoxyphenyl)benzo[d]oxazole* (**7**). Aldehyde **6** (3.46 g, 16.1 mmol), 2-aminophenol (1.93 g, 17.1 mmol), and NaCN (0.039 g, 0.804 mmol) were transferred to a 100 mL round-bottom flask. The reagents were dissolved in 60 mL of DMF, and an oxygen balloon was attached to the reaction mixture. The mixture was stirred at 50 °C for 4 h and monitored by TLC. Upon completion of the reaction, the mixture was evaporated, diluted with saturated Na_2_CO_3_, and extracted with DCM (3 × 25mL). The organic layer was dried over Na_2_SO_4_ and concentrated. The product was purified using flash column chromatography (silica gel, EtOAc/hexanes, from 1:19 to 1:9). The product was recrystallized from cold EtOH to afford 3.33 g (68%) of compound **7** as light-yellow needles. ^1^H-NMR (600 MHz, CDCl_3_) δ 8.28 (d, *J* = 2.6 Hz, 1H), 7.85–7.79 (m, 1H), 7.61–7.57 (m, 2H), 7.38–7.35 (m, 2H), 6.97 (d, *J* = 8.9 Hz, 1H), 4.01 (s, 3H). ^13^C-NMR (151 MHz, CDCl_3_) δ 160.0, 157.5, 150.3, 141.9, 135.2, 133.6, 125.3, 124.5, 120.4, 117.9, 113.9, 112.8, 110.5, 56.5. HRMS (EI *m*/*z*): [M^+•^] Calcd. for C_14_H_10_BrNO_2_: 302.9895; found, 302.9889.

*2-(2-Methoxy-4-(4,4,5,5-tetramethyl-1,3,2-dioxaborolan-2-yl)phenyl)benzo[d]oxazole* (**8**). Compound **7** (502 mg, 1.65 mmol), bis(pinacolato)diboron (461 mg, 1.81 mmol) and potassium acetate (486 mg, 1.81 mmol) in 20 mL of 1,4-dioxane were placed in a dry, argon-purged Schlenk tube. Next, Pd(dppf)Cl_2_ was added to the mixture, heated to 100 °C, and stirred overnight. The mixture was quenched with EtOAc and filtered through short pad of Celite. The product was purified using flash column chromatography (silica gel; EtOAc/hexanes, 2:3) and recrystallized from cold EtOH to afford 463 mg (80%) of compound **8** as light-yellow needles. ^1^H-NMR (400 MHz, CDCl_3_) δ 8.15 (d, *J* = 7.7 Hz, 1H), 7.86–7.80 (m, 1H), 7.62–7.57 (m, 1H), 7.54 (d, *J* = 7.7 Hz, 1H), 7.50 (s, 1H), 7.38–7.30 (m, 2H), 4.07 (s, 3H), 1.37 (s, 12H). ^13^C-NMR (101 MHz, CDCl_3_) δ 161.5, 157.7, 150.4, 142.2, 130.4, 127.0, 125.0, 124.3, 120.3, 118.5, 117.7, 110.5, 84.2, 56.3, 24.9. HRMS (EI *m*/*z*): [M^+•^] Calcd. for C_20_H_22_BNO_4_: 351.1642; found, 351.1648.

*2-(5-Bromo-2-methoxyphenyl)benzo[d]oxazole* (**10**). Compound **9** (2.00 g, 9.3 mmol), 2-aminophenol (1.68 g, 15.3 mmol), and NaCN (0.022 g, 0.465 mmol) were transferred to a 100 mL round-bottom flask. The reagents were dissolved in 40 mL of DMF. An oxygen balloon was attached, and the reaction mixture was stirred at 110 °C for 2 h. Upon completion of the reaction, the volatiles were evaporated. The product was purified using flash column chromatography (silica gel; EtOAc/hexanes, 3:17) and recrystallized from cold EtOH to afford 1.94 g (68%) of compound **10** as light-yellow needles. ^1^H-NMR (600 MHz, CDCl_3_) δ 8.28 (d, *J* = 2.6 Hz, 1H), 7.83 (ddd, *J* = 5.7, 2.2, 0.7 Hz, 1H), 7.63–7.56 (m, 2H), 7.42–7.32 (m, 2H), 6.98 (d, *J* = 8.9 Hz, 1H), 4.01 (s, 3H). ^13^C-NMR (151 MHz, CDCl_3_) δ 160.0, 157.5, 150.3, 141.8, 135.3, 133.6, 125.4, 124.5, 120.4, 117.9, 113.9, 112.8, 110.5, 56.5. HRMS (EI *m*/*z*): [M^+•^] Calcd. for C_14_H_10_BrNO_2_: 302.9895; found, 302.9904.

*2-(2-Methoxy-5-(4,4,5,5-tetramethyl-1,3,2-dioxaborolan-2-yl)phenyl)benzo[d]oxazole* (**11**). Compound **10** (502 mg, 1.65 mmol), bis(pinacolato)diboron (461 mg, 1.81 mmol), and potassium acetate (486 mg, 1.81 mmol) in 20 mL of 1,4-dioxane were placed into a dry, argon-purged Schlenk tube. Next, Pd(dppf)Cl_2_ was added to the mixture and was heated at 100 °C overnight. After completion of the reaction, it was quenched with EtOAc and filtered through short pad of Celite. The product was purified using flash column chromatography (silica gel; EtOAc/hexanes, 3:7) and recrystallized from cold EtOH to afford 475 mg (82%) of compound **11** as light-yellow needles. ^1^H-NMR (600 MHz, CDCl_3_) δ 8.58 (d, *J* = 1.7 Hz, 1H), 7.95 (dd, *J* = 8.4, 1.7 Hz, 1H), 7.86–7.80 (m, 1H), 7.62–7.57 (m, 1H), 7.37–7.31 (m, 2H), 7.08 (d, *J* = 8.4 Hz, 1H), 4.05 (s, 3H), 1.36 (s, 12H). ^13^C-NMR (151 MHz, CDCl_3_) δ 161.4, 160.8, 150.2, 142.1, 139.6, 138.1, 124.9, 124.2, 120.2, 115.6, 111.3, 110.4, 83.9, 75.0, 56.2, 24.9. HRMS (EI *m*/*z*): [M^+•^] Calcd. for C_20_H_22_BNO_4_: 351.1642; found, 351.1649.

*3,6-Bis(5-(4-(benzo[d]oxazol-2-yl)-3-methoxyphenyl)thiophen-2-yl)-2,5-dihexylpyrrolo[3,4-c]pyrrole-1,4(2H,5H)-dione* (**13**). Compound **12** (187 mg, 0.40 mmol), aryl bromide **7** (280 mg, 0.92 mmol), palladium (II) acetate (5.0 mg, 5 mol%), and potassium acetate (86 mg, 0.88 mmol) were placed in a dry, argon-purged Schlenk tube. DMA (3 mL) was added, and the suspension was degassed 3 times by evacuation, and the Schlenk tube was refilled with argon gas. The mixture was heated at 150 °C overnight. The product was purified using flash column chromatography (silica gel; with gradient from CHCl_3_ to CHCl_3_/EtOAc, 1:4) and recrystallized from EtOAc to afford 120 mg (33%) of **13** as violet shiny crystals. ^1^H-NMR (500 MHz, CDCl_3_ + TFA-*d*_1_) δ 8.78 (br s, 2H), 8.53 (br s, 2H), 8.08 (d, *J* = 11.9 Hz, 2H), 7.96 (d, *J* = 9.1 Hz, 2H), 7.87 (d, *J* = 8.9, 2H), 7.74–7.65 (m, 4H), 7.58 (d, *J* = 4.1 Hz, 2H)), 7.33 (d, *J* = 8.9 Hz, 2H), 4.19 (s, 6H), 4.17–4.12 (m, 4H), 1.86–1.76 (m, 4H), 1.51–1.43 (m, 4H), 1.41–1.32 (m, 8H), 0.91 (t, *J* = 6.9 Hz, 6H). ^13^C-NMR (126 MHz, CDCl_3_) δ 162.1, 160.8, 160.2, 147.7, 147.6, 143.2, 141.2, 137.0, 131.7, 131.2, 129.4, 129.1, 128.5, 120.0, 116.0, 112.2, 110.0, 109.4, 109.3, 107.2, 57.0, 43.0, 31.2, 29.7, 26.4, 22.4, 13.8. HRMS (EI *m*/*z*): [M^+•^] Calcd. for C_54_H_50_N_4_O_6_S_2_: 914.3172; found, 914.3168.

*3,6-Bis(5-(3-(benzo[d]oxazol-2-yl)-4-methoxyphenyl)thiophen-2-yl)-2,5-dihexylpyrrolo[3,4-c]pyrrole-1,4(2H,5H)-dione* (**15**). Dye **11** (176 mg, 0.5 mmol), compound **14** (76 mg, 0.25 mmol), Cs_2_CO_3_ (489 mg, 1.5 mmol), and Pd(dppf)Cl_2_ (18.3 mg, 0.025 mmol) were suspended in a dry toluene (2 mL) in an argon-purged Schlenk tube. The mixture was heated at 107 °C overnight. The reaction mixture was concentrated, and the product was purified by flash column chromatography (silica gel; with gradient: from CHCl_3_ to CHCl_3_/EtOAc, 1:4). The product was recrystallized from MeOH to afford 55 mg (24%) of **15** as dark purple solid. ^1^H-NMR (500 MHz, CDCl_3_) δ 8.70 (d, *J* = 4.0 Hz, 2H), 8.59 (d, *J* = 2.4 Hz, 2H), 8.15 (m, 2H), 7.92 (m, 4H), 7.77 (m, 4H), 7.63 (d, *J* = 4.0 Hz, 2H), 7.39 (d, *J* = 8.9 Hz, 2H), 4.23 (s, 6H), 4.15 (t, *J* = 8.0 Hz, 4H), 1.82 (quin, *J* = 7.9 Hz, 4H), 1.48 (qiun, *J* = 7.4, 4H), 1.42–1.32 (m, 8H), 0.91 (t, *J* = 6.9 Hz, 6H). ^13^C-NMR (126 MHz, CDCl_3_) δ 162.1, 160.5, 160.1, 147.9, 147.7, 141.3, 137.2, 136.9, 129.7, 129.2, 128.8, 128.5, 127.9, 127.6, 126.0, 116.4, 113.9, 112.2, 108.4 (2 signals), 57.3, 31.3, 29.7, 29.7, 26.4, 22.5, 13.9. HRMS (EI *m*/*z*): [M^+•^] Calcd. for C_54_H_50_N_4_O_6_S_2_: 914.3172; found, 914.3146.

All density functional theory (DFT) and time-dependent DFT (TD-DFT) calculations were carried out with the Gaussian 16 package [97]. The integral equation formalism (IEF) version of the polarizable continuum (PCM) model was used to include the solvent effects, considering DCM (*ε* = 8.93) as medium. The ground state geometries of **5**, **13**, and **15** were first fully optimized using the M06-2X [98] exchange-correlation functional with the 6-311G (d,p) atomic basis set. Next, frequency calculations were performed at the same level of theory to ensure that each optimized structure was a true minimum on the potential energy surface (absence of imaginary frequency). The geometries of the lowest electronic excited states of the three DPP dyes were similarly obtained at the TD-M06-2X/6-311G(d,p) level of theory, and analytic frequency calculations were also done at the same level to check that their excited states structures were true minima of the excited state potential energy surface. From the optimal equilibrium geometries, the vertical transition energies were calculated using the same hybrid functional but a larger basis set—namely, 6-311+G(2d,p). The solvent effects were accounted for using the linear-response PCM model, adequate for bright transitions.

## 5. Conclusions

There are multiple implications of the results of this study. The synthesis of 2,5-diaryl-diketopyrrolopyrroles, possessing complex substituents, could be realized with the help of direct arylation. The presence of thiophene moiety directly linked with diketopyrrolopyrrole core enables a significant bathochromic shift of both absorption and emission. The presence of substituents fails to impose any charge-transfer character on the S_1_ state. The peripheral aryl substituents are inactive in the S_0_→S_1_ transition, resulting in a small influence on the type of substitution of the photophysical properties. Finally, shifting the benzoxazole moiety in diketopyrrolopyrroles changes the principal deactivation pathway, with intersystem crossing prevailing when all units are bridged in *para* positions. These findings may serve as a blueprint to design diketopyrrolopyrroles with fine-tuned optical properties.

## Figures and Tables

**Table 1:** Photophysical properties of DPPs **5**, **13**, and **15**.

DPP	Solvent	λ_abs_ (nm)	λ_em_(nm)	Φ_fl_	τ (ns)	*k_f_* × 10^7^/s^−1^	*k_nr_* × 10^7^/s^−1^
**5**	TOL	528	593	0.89	4.16	21.1	2.95
	DCM	515	589	0.86	4.35	19.7	3.27
	DMF	512	591	0.72	4.39	16.0	6.09
**13**	TOL	622	655	0.31	2.87	10.8	24.1
	DCM	614	648	0.24	2.94	8.23	25.8
	DMF	619	645	0.25	2.71	9.83	27.6
**15**	TOL	611	635	0.34	3.35	10.1	19.7
	DCM	606	633	0.32	3.23	9.92	21.0
	DMF	610	633	0.19	3.10	6.26	26.0

## Data Availability

The data presented in this study are available in this article and accompanying supplementary material.

## Supplementary Materials

The following are available online. Figure S1: ^1^H and ^13^C-NMR spectral data for **4**, Figure S2: ^1^H and ^13^C-NMR spectral data for **5**, Figure S3: ^1^H and ^13^C-NMR spectral data for **7**, Figure S4: ^1^H and ^13^C-NMR spectral data for **8**, Figure S5: ^1^H and ^13^C-NMR spectral data for **10**, Figure S6: ^1^H and ^13^C-NMR spectral data for **11**, Figure S7: ^1^H and ^13^C-NMR spectral data for **13**, Figure S8: ^1^H and ^13^C-NMR spectral data for **15**.Figure S9: Absorption and emission spectra of DPPs **5**, **13** and **15** in toluene, Figure S10: Absorp-tion and emission spectra of DPPs **5**, **13** and **15** in DMF.

## Sample Availability

Samples of the compounds **5**, **13**, and **15** are available from the authors.
