# Supplementary material for: The Synthesis and Photophysical Properties of Weakly Coupled Diketopyrrolopyrroles
Source: Molecules. 2021 Aug 5;26(16):4744. doi: 10.3390/molecules26164744 (PMC8398321; doi:10.3390/molecules26164744)
Supplement: Supplementary file 1 [file molecules-26-04744-s001.zip › molecules-1313605-supplementary.pdf]

molecules

MDPI

Supplementary Material

The Synthesis and Photophysical Properties of Weakly Coupled Diketopyrrolopyrroles

Michał Pieczykolan [1], James B. Derr [2], Amara Chrayteh [3], Beata Koszarna [1], John A. Clark [4], Olena Vakuliuk [1], Denis Jacquemin [3,*], Valentine I. Vullev [2,4,*] and Daniel T. Gryko [1,*]

[1] Institute of Organic Chemistry, Polish Academy of Sciences, Kasprzaka 44-52, 01-224 Warsaw, Poland; Michal.Pieczykolan@rwth-aachen.de (M.P.); beata.koszarna@icho.edu.pl (B.K.); olena.vakuliuk@icho.edu.pl (O.V.)

[2] Department of Biochemistry, University of California Riverside, Riverside, CA 92521, USA; jderr002@ucr.edu (J.B.D.); jclar019@ucr.edu (J.A.C.)

[3] CEISAM Laboratory—UMR 6230, University of Nantes, CNTS, 44035 Nantes, France; amara.chrayteh@univ-nantes.fr

[4] Department of Bioengineering, University of California Riverside, Riverside, CA 92521, USA

* Correspondence: Denis.Jacquemin@univ-nantes.fr (D.J.); vullev@ucr.edu (V.I.V.); dtgryko@icho.edu.pl (D.T.G.)

Table of contents

Figure S1: ^{1}H and ^{13}C NMR spectral data for **4** *S2*
Figure S2: ^{1}H and ^{13}C NMR spectral data for **5** *S3*
Figure S3: ^{1}H and ^{13}C NMR spectral data for **7** *S4*
Figure S4: ^{1}H and ^{13}C NMR spectral data for **8** *S5*
Figure S5: ^{1}H and ^{13}C NMR spectral data for **10** *S6*
Figure S6: ^{1}H and ^{13}C NMR spectral data for **11** *S7*
Figure S7: ^{1}H and ^{13}C NMR spectral data for **13** *S8*
Figure S8: ^{1}H and ^{13}C NMR spectral data for **15** *S9*
Figure S9: Absorption and emission spectra of DPPs **5**, **13** and **15** in toluene *S10*
Figure S10: Absorption and emission spectra of DPPs **5**, **13** and **15** in DMF *S10*

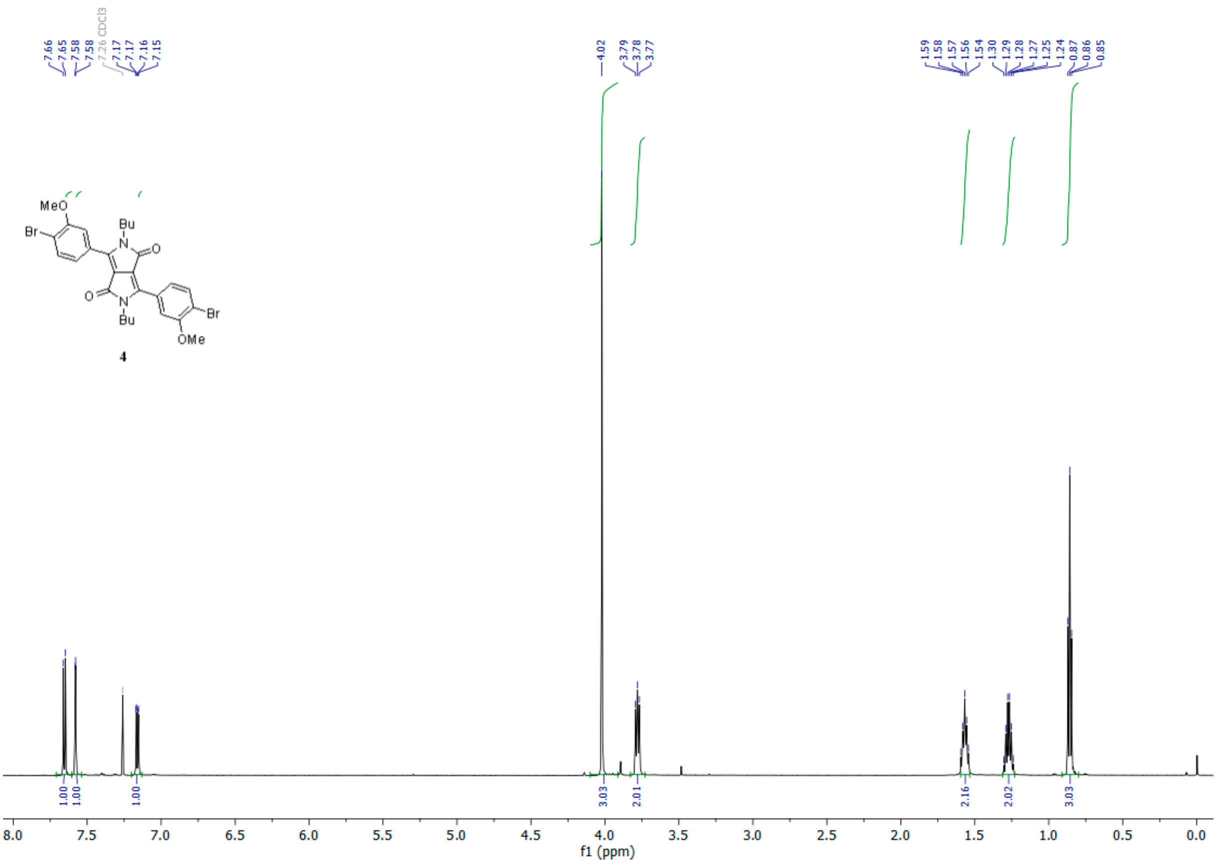

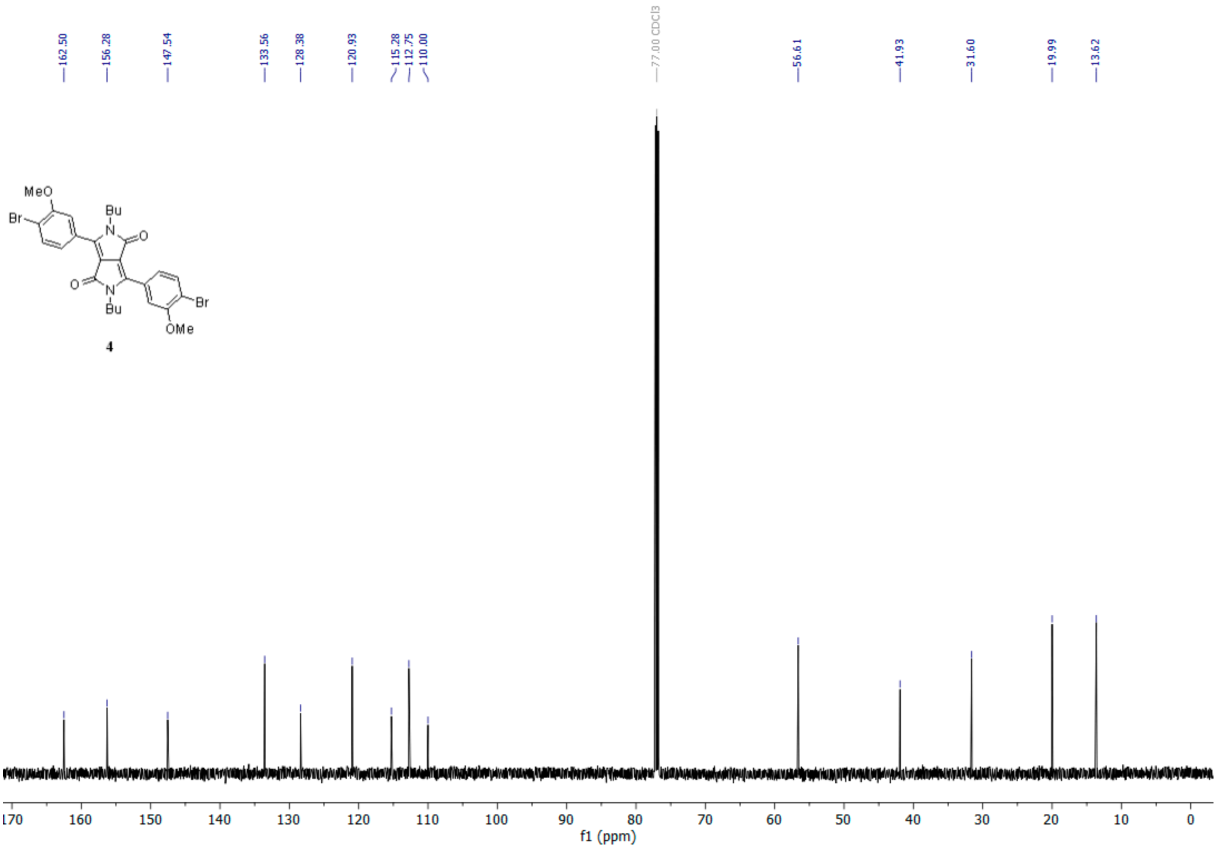

Figure S1. ^{1}H and ^{13}C-NMR spectral data for **4.**

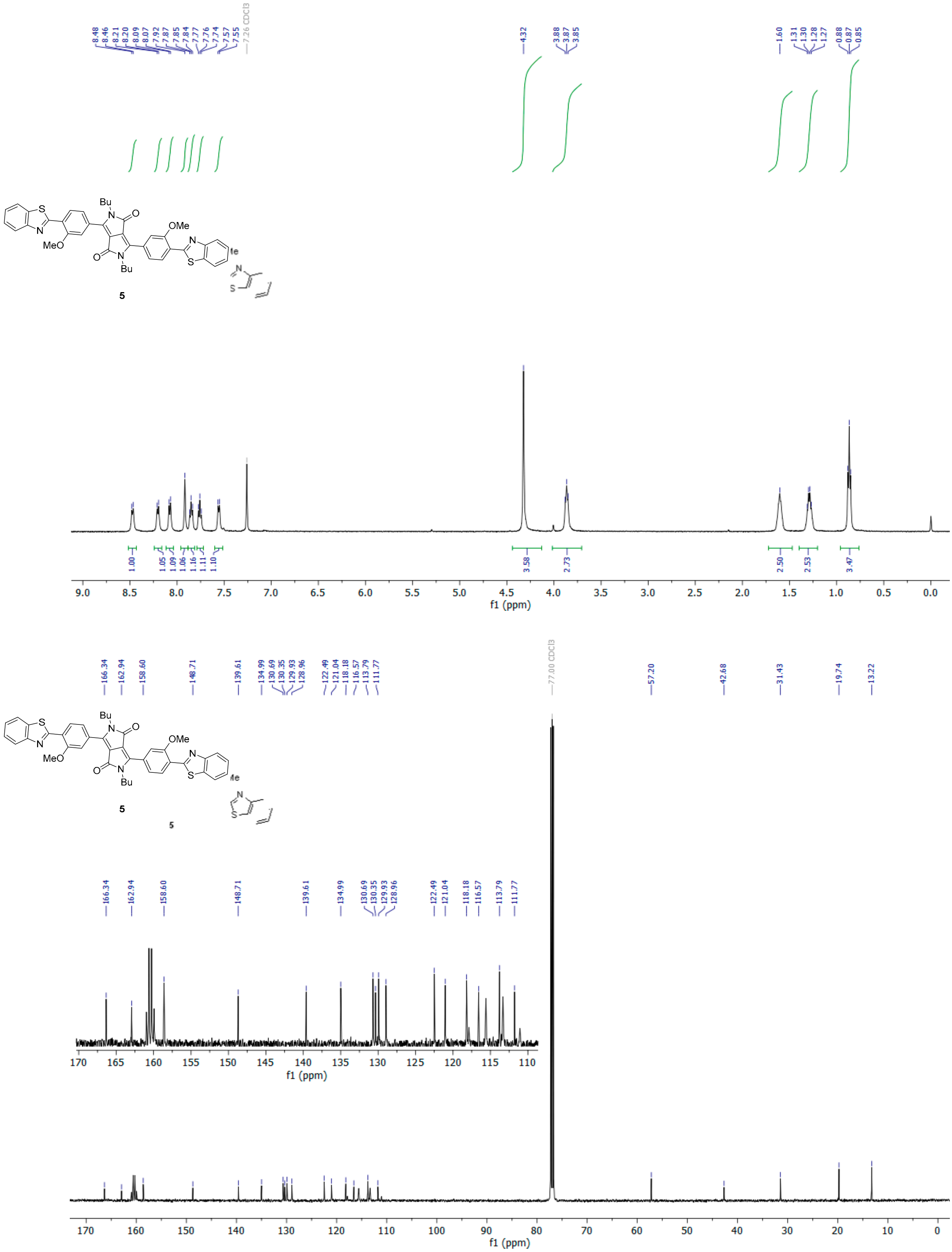

Figure S2. ^{1}H and ^{13}C-NMR spectral data for **5.**

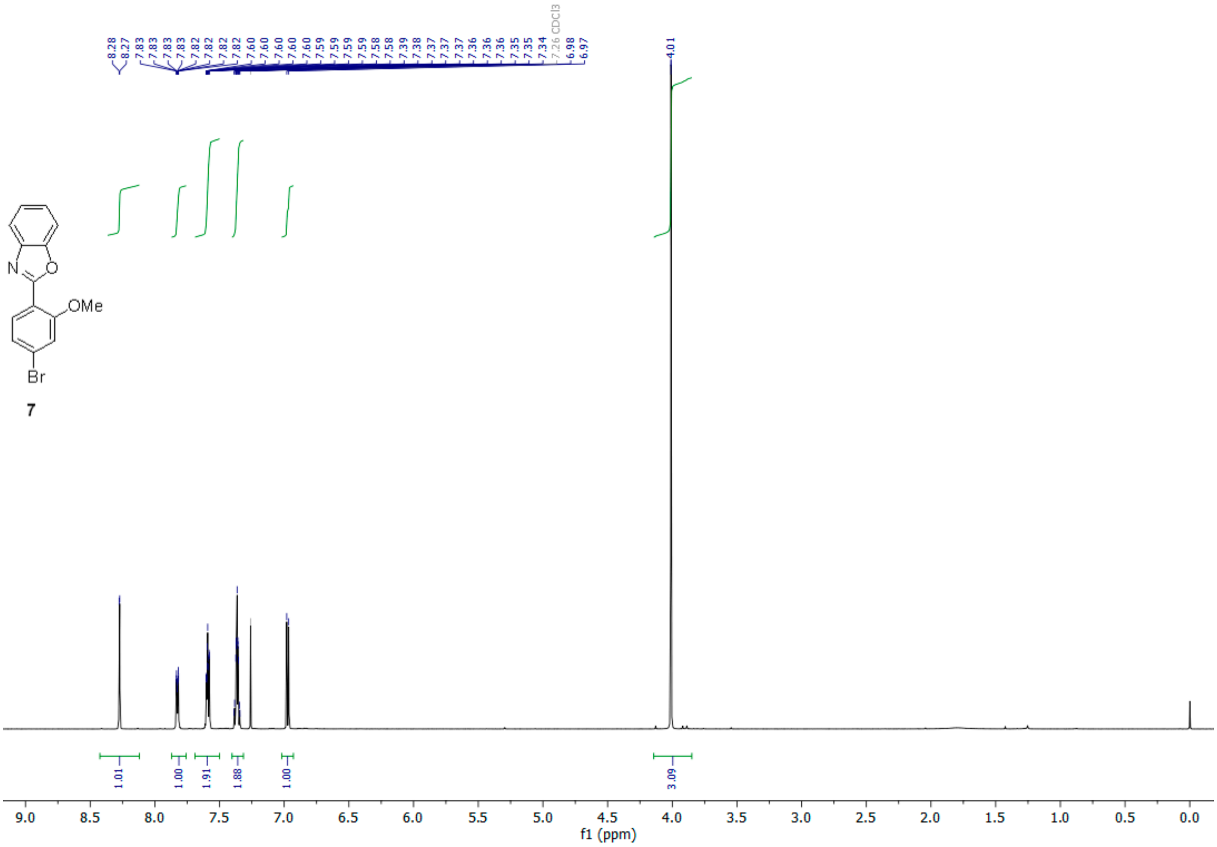

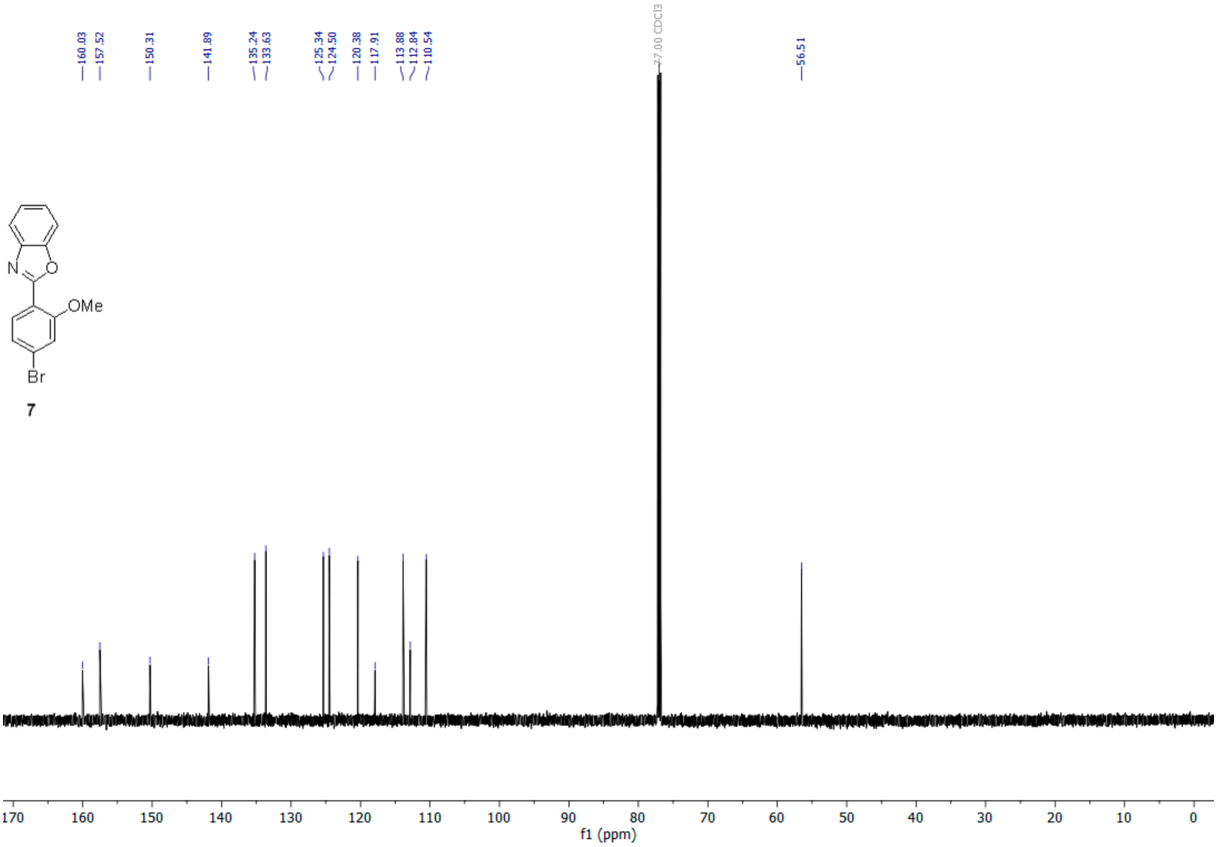

Figure S3. ^{1}H and ^{13}C-NMR spectral data for **7.**

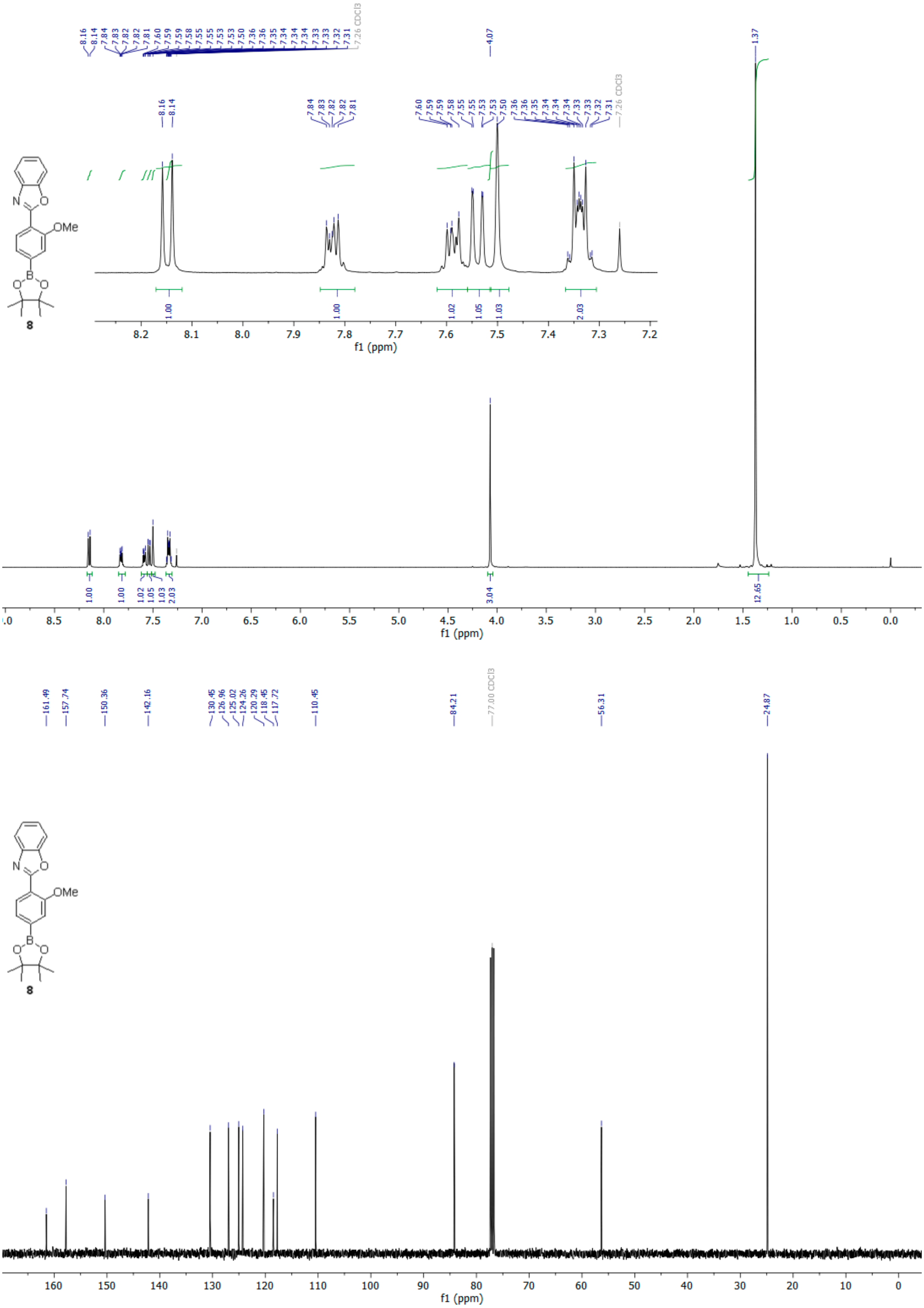

Figure S4. ^{1}H and ^{13}C-NMR spectral data for **8.**

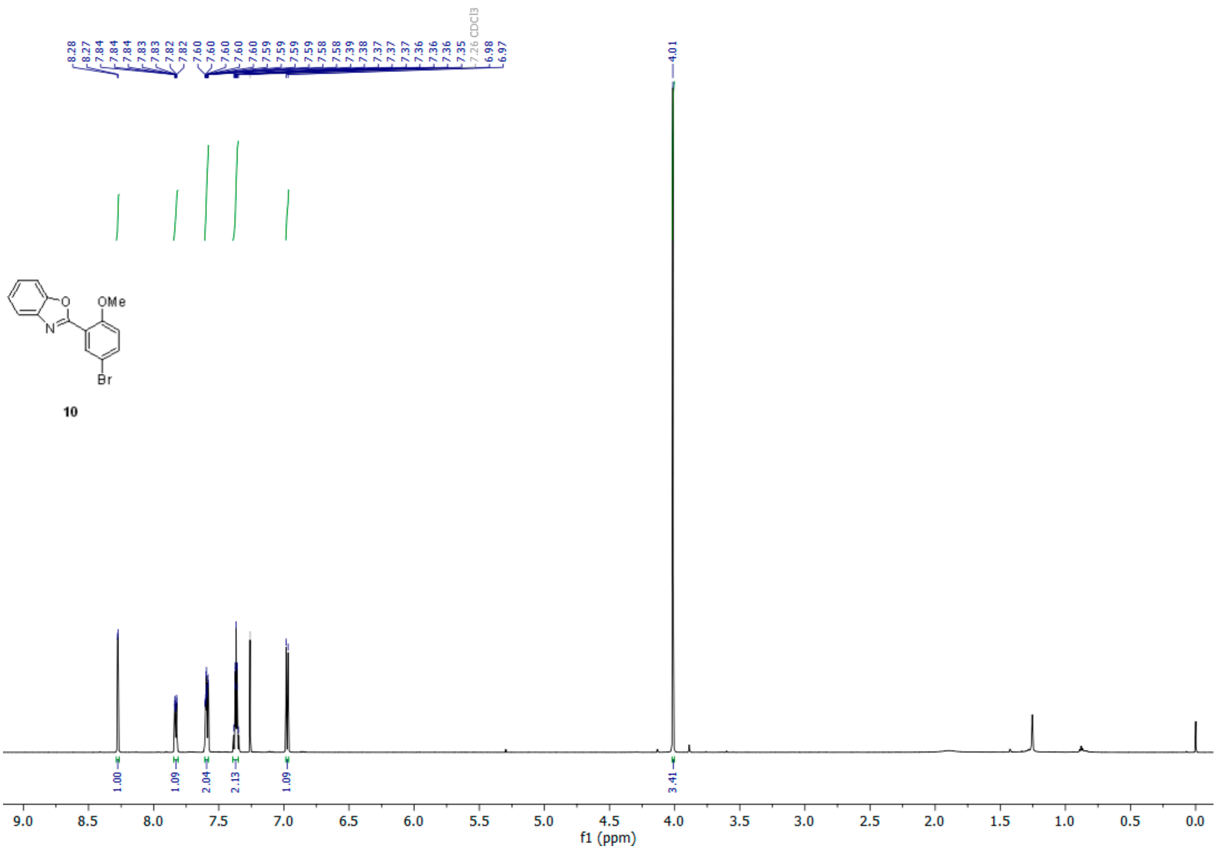

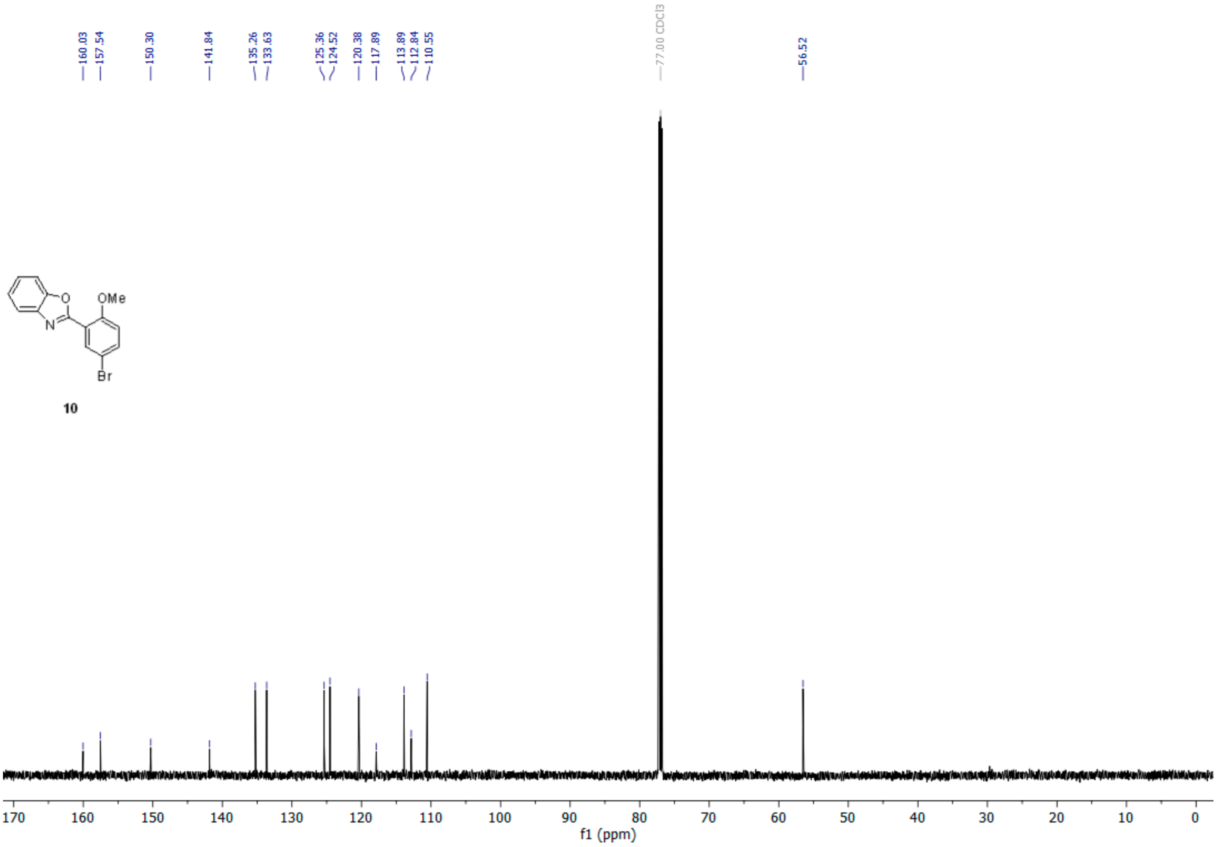

Figure S5. ^{1}H and ^{13}C-NMR spectral data for **10.**

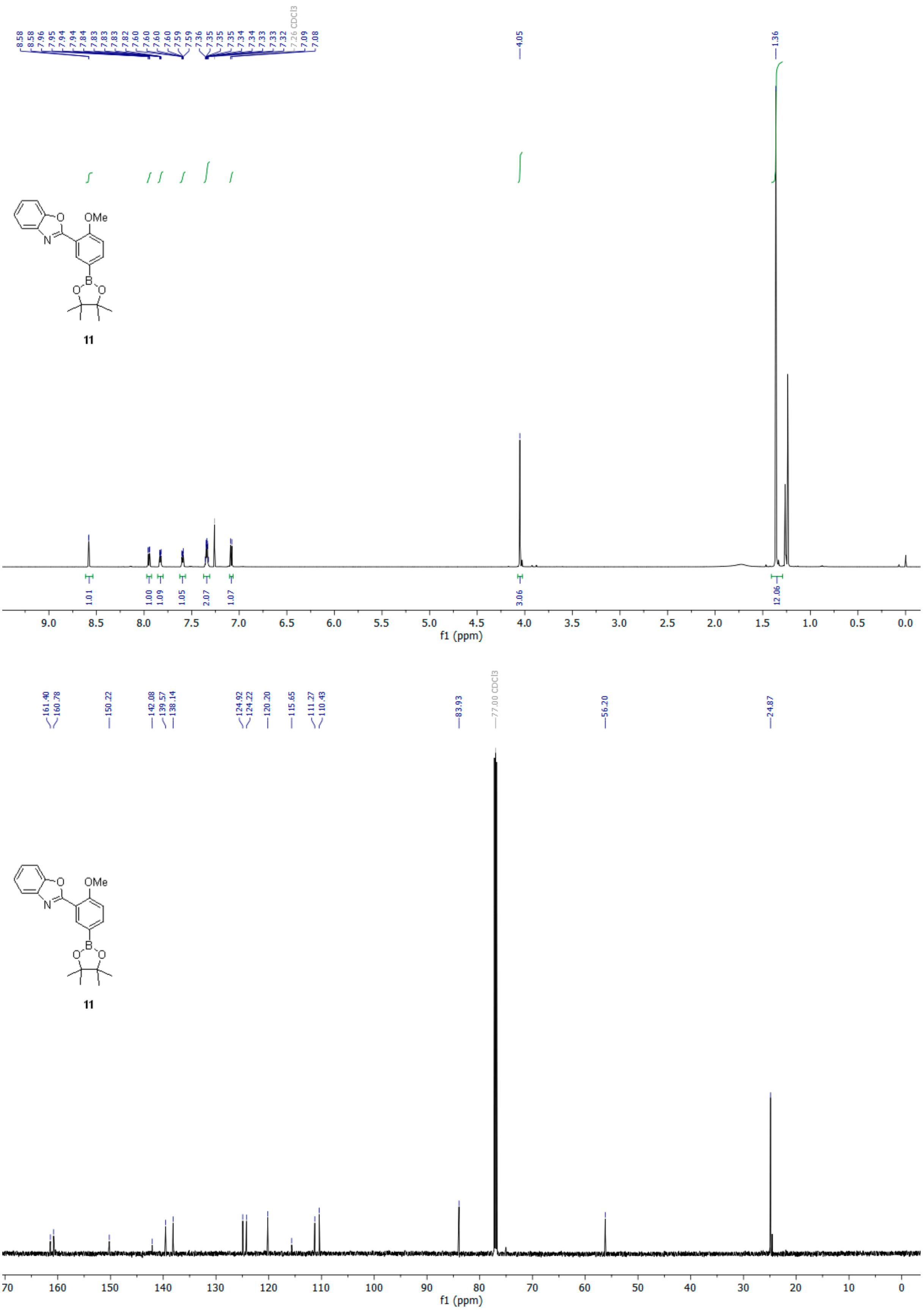

Figure S6. ^{1}H and ^{13}C-NMR spectral data for **11.**

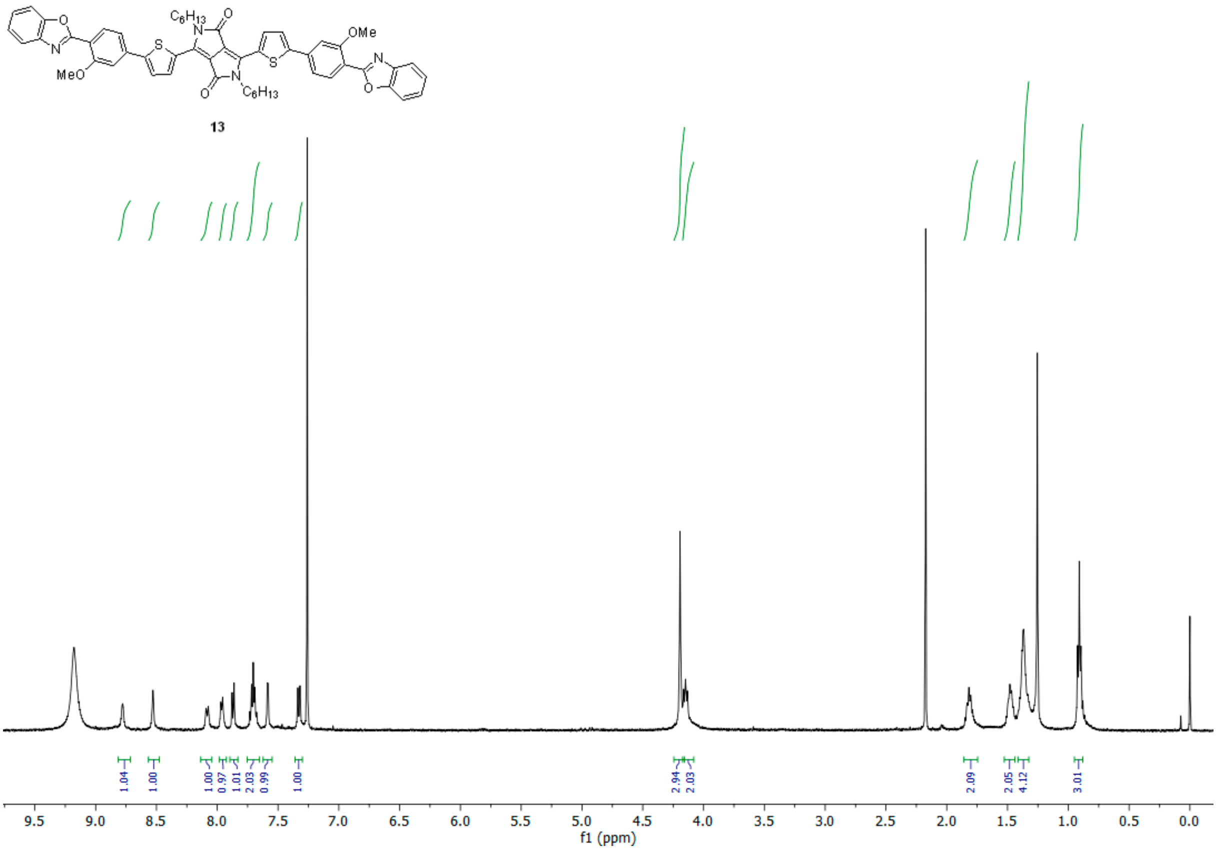

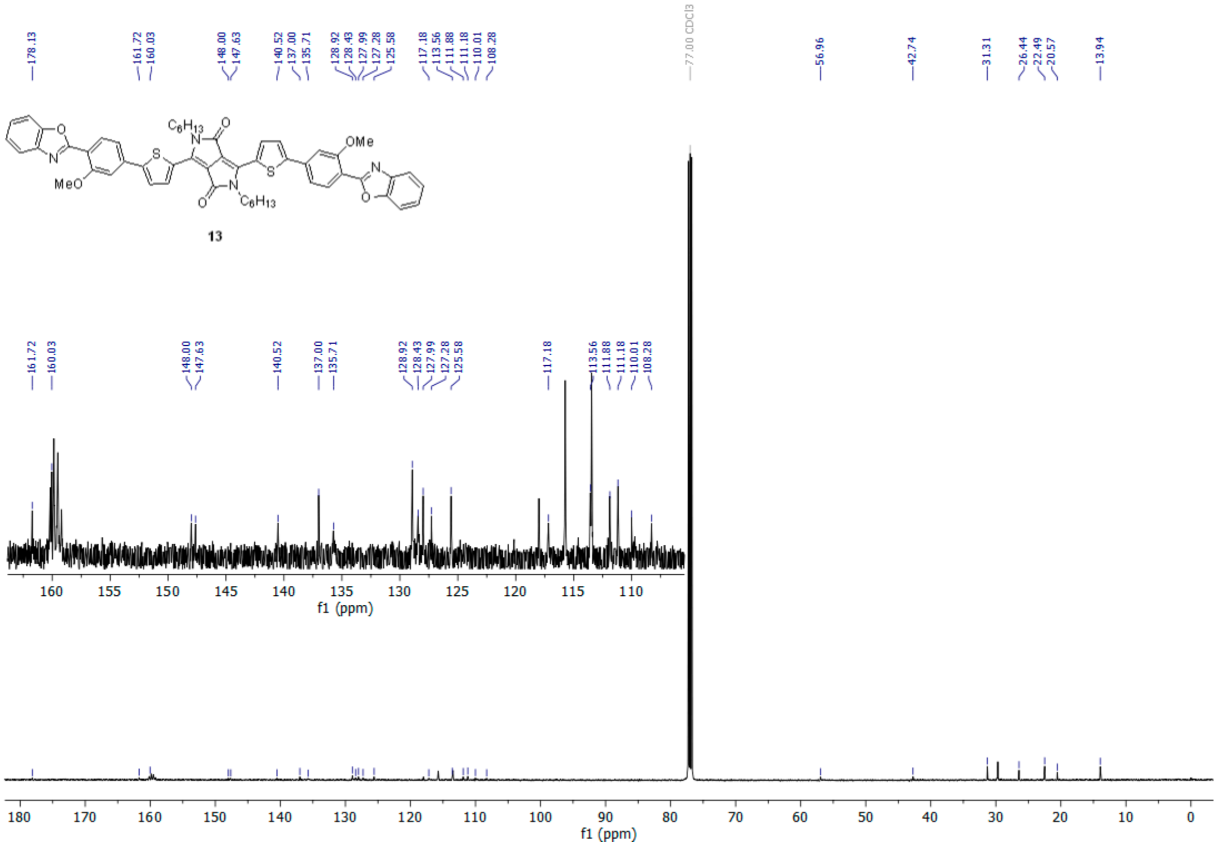

Figure S7. ^{1}H and ^{13}C-NMR spectral data for **13.**

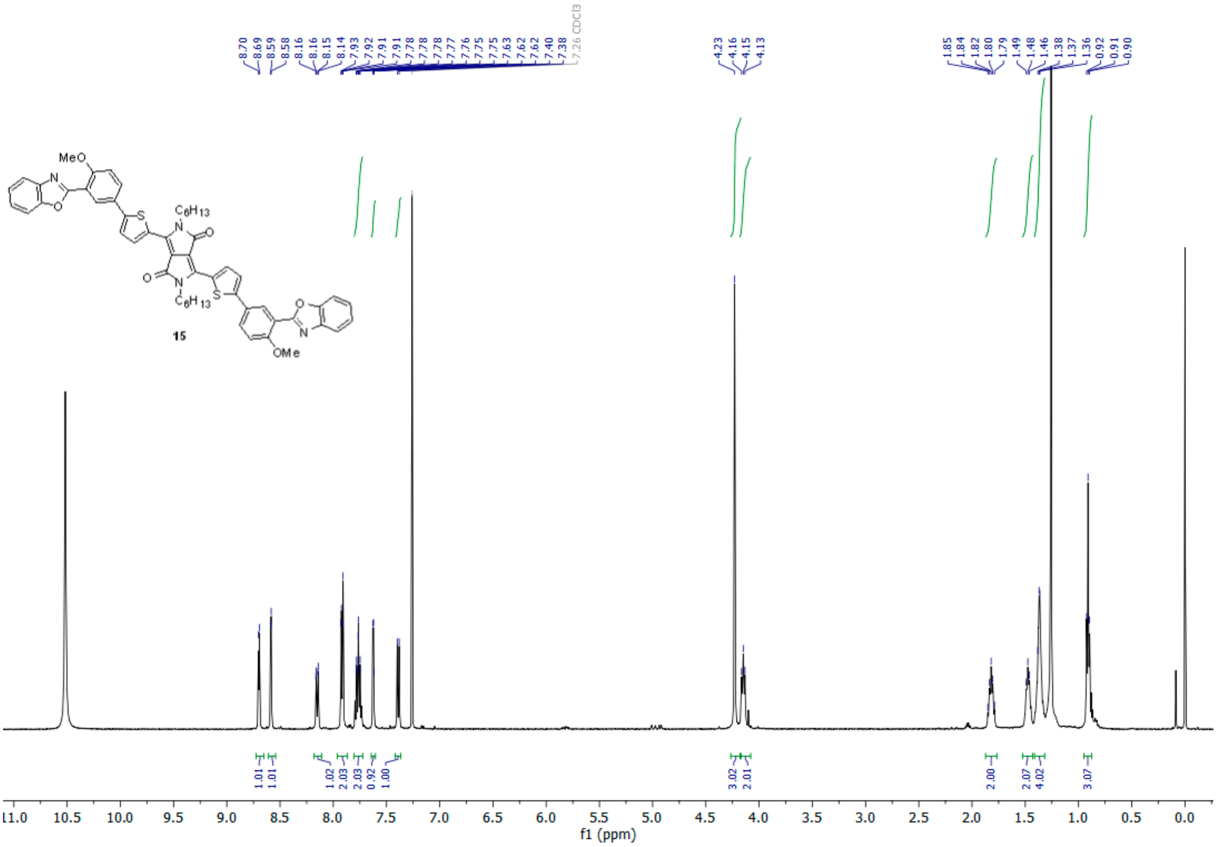

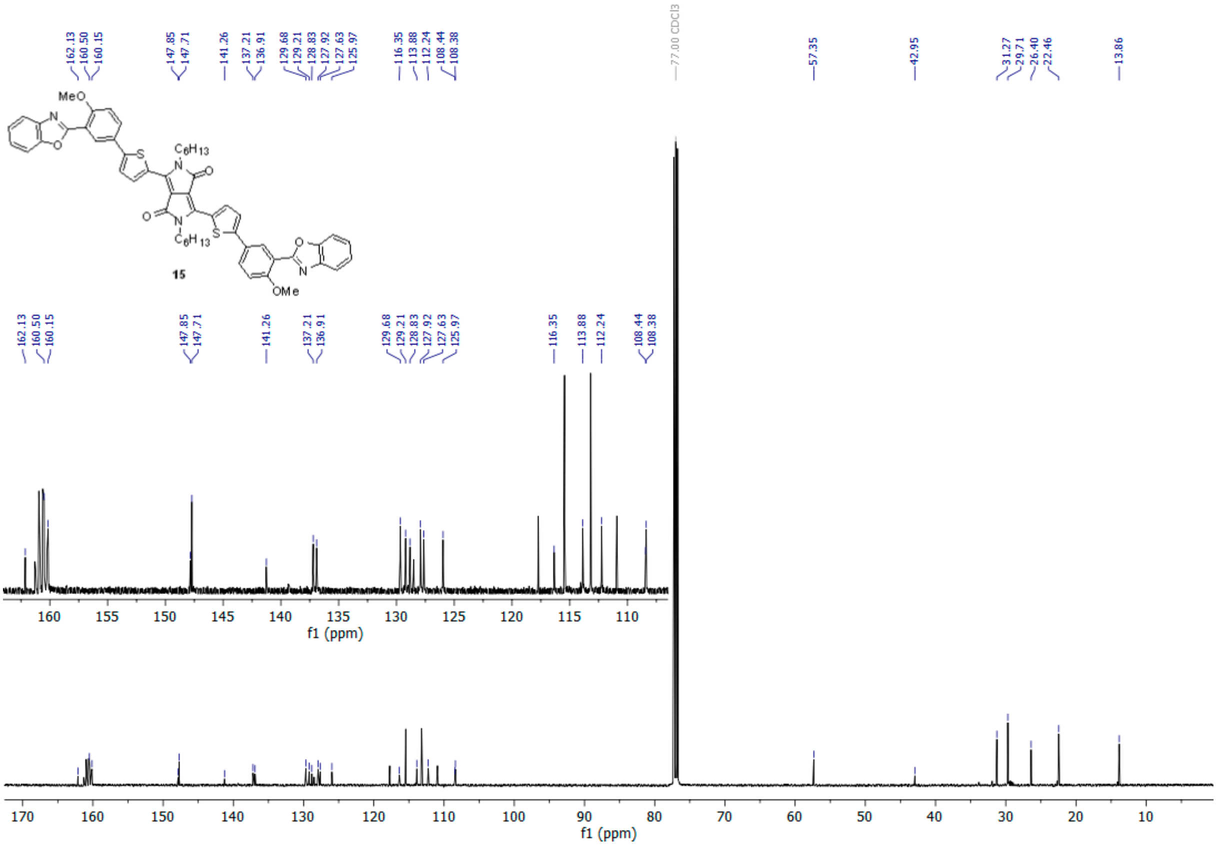

Figure S8. ^{1}H and ^{13}C-NMR spectral data for **15.**

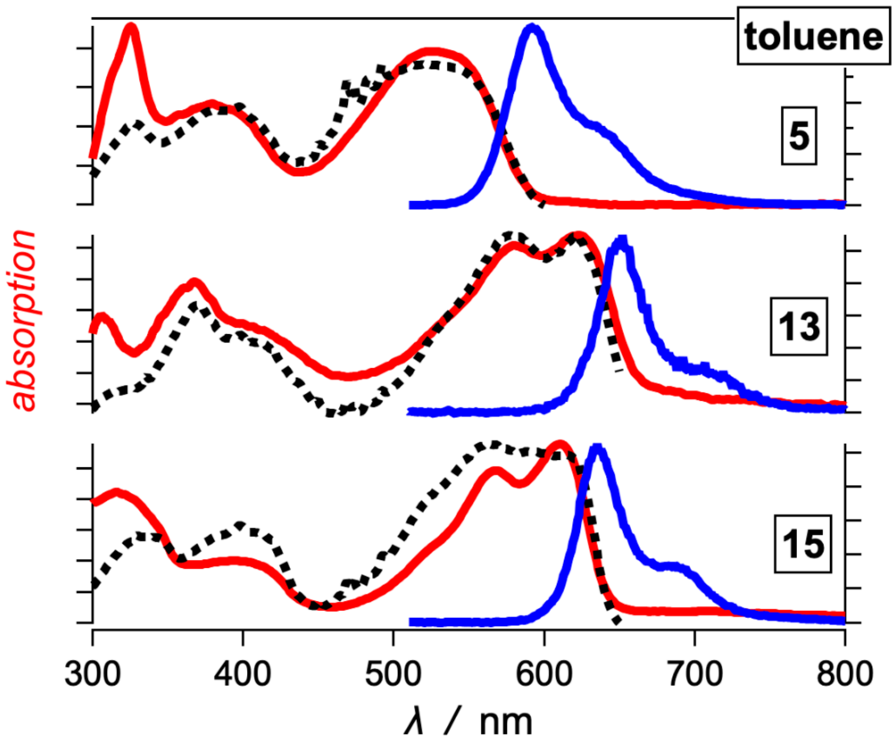

Figure S9. Absorption (red), excitation (dotted black), and emission (blue) spectra of DPPs **5**, **13** and **15** in toluene.

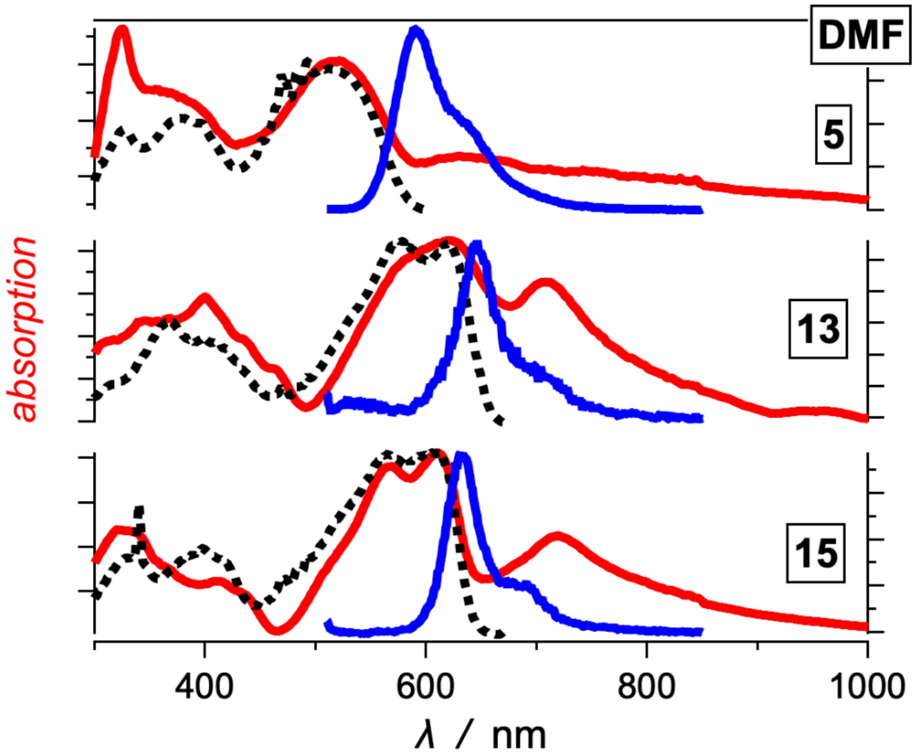

Figure S10. Absorption (red), excitation (dotted black), and emission (blue) spectra of DPPs **5**, **13** and **15** in DMF.
